# Nutritional status of school age children in Abakaliki metropolis, Ebonyi State, Nigeria

**DOI:** 10.1186/s12887-020-1994-5

**Published:** 2020-03-07

**Authors:** Adanna Anthonia Umeokonkwo, Maryann Ugochi Ibekwe, Chukwuma David Umeokonkwo, Clifford Onuorah Okike, Obumneme Benaiah Ezeanosike, Bede Chidozie Ibe

**Affiliations:** 1 Department of Paediatrics, Alex Ekwueme Federal University Teaching Hospital, Abakaliki, Ebonyi State Nigeria; 2 Department of Community Medicine, Alex Ekwueme Federal University Teaching Hospital, Abakaliki, Ebonyi State Nigeria; 3 Department of Paediatrics, University of Nigeria Teaching Hospital Ituku Ozalla, Enugu, Enugu State Nigeria

**Keywords:** Stunting, Underweight, Thinness, BMI, Malnutrition, Overweight

## Abstract

**Background:**

Malnutrition is a major public health problem with short and long-term adverse effects on children particularly in middle and low-income countries. Three out of every ten under-five children are said to be stunted and 19.4% underweight in Nigeria. In Ebonyi State, between 2013 and 2015, the proportion of preschool children with chronic undernutrition rose from 16.2 to 20.6%. Little is documented about the nutritional status of school-age children in Ebonyi State and Nigeria.

**Methods:**

We conducted a descriptive cross-sectional study among 780 children aged 6 to 12 years from 10 primary schools in Abakaliki metropolis. A multistage sampling method was used to select the participants. A pre-tested interviewer-administered structured questionnaire was used to collect information from the children and their parents. Body Mass Index (BMI), Z scores of the weight for age, BMI for age and height for age were obtained using the WHO AnthroPlus software. We estimated the prevalence of undernutrition, over-nutrition, underweight, thinness, stunting, overweight and obesity.

**Result:**

Out of 751 pupils that participated, 397 (52.9%) were females and 595 (79.2%) were in public schools. The overall prevalence of undernutrition was 15.7% and that of over-nutrition was 2.1%. The prevalence of underweight, thinness and stunting, overweight and obesity were 8, 7.2, 9.9, 1.4 and 0.7% respectively. The proportion of pupils who were thin was higher among males (8.7%), those attending public schools (8.6%) and those dwelling in rural parts of the metropolis (14.3%) compared to females (5.8%) private school attendees (1.9%) and urban dwellers (4.6%). Stunting was found to be higher among pupils attending public schools (11.8%) compared to those attending private schools (2.5%). The prevalence of stunting was 19.3% among the pupils residing in rural areas and 5% among the pupils living in urban areas of the metropolis. No pupil in private schools was underweight. Over-nutrition was not found among the pupils in rural areas.

**Conclusion:**

Both under and over nutrition exist in Abakaliki metropolis. Undernutrition is the more prevalent form of malnutrition among school age children in the metropolis.

## Background

Malnutrition is a spectrum that include both under or over-nutrition. Undernutrition occurs as a result of inadequate food intake or poor absorption (due to disease) and has negative impact on growth and development [1–4]. Undernutrition is the most prevalent form of malnutrition in resource-poor countries and is comprised of stunting, thinness, and underweight [5]. Undernutrition has been defined as height for age, weight for age, weight for height or body mass index for age, below − 2 Z score of the mean value for age and sex [5–8]. Overnutrition is defined as BMI for age greater than + 1 Z score for age and sex [9].

Undernutrition is a major public health problem worldwide, especially in low and middle-income countries [2, 10]. Globally, in 2015, 159 million children under the age of five were estimated to be stunted and more than a third of these live in Africa [11, 12]. Fifty million under-five children were wasted with 16 million being severely wasted, in 2015 [11]. In Nigeria, 33% of under-five children were stunted, 19.4% were underweight while 7.2% had moderate acute malnutrition in 2015 [13]. Undernutrition accounted for about half of all childhood deaths in Nigeria [14]. In Ebonyi State, between 2013 and 2015, the proportion of children under five who were chronically undernourished rose from 16.2 to 20.6%, in separate national surveys [13, 15]. The prevalence of stunting (20.6%) was the highest among the Southeastern States of Nigeria [13].

Undernutrition causes immune dysfunction with an increased risk of infection [16] cognitive impairment [4, 17, 18], reduced physical capacity [4, 17] and growth faltering [19, 20]. An undernourished child has a higher risk of neurocognitive delay, poor school performance, early school dropout [16] and therefore is more likely to have reduced work productivity, earning potentials and will contribute less to the nation’s Gross Domestic Product(GDP) later in life [18]. In women, stunting increases the risk of mortality during childbirth and leads to adverse birth outcomes [21]. Global Nutrition Report(GNR) estimates that malnutrition causes a huge economic loss of about 11% of Gross Domestic Product per year in Africa and Asia [12].

Previous studies done on malnutrition focused on under 5 (preschool) children with emphasis on intervention within the first one thousand days of life [17, 19]. School age is a period of rapid and active growth and developmental changes [16, 22–24], which inadequate diet and unfavourable environment may adversely affect. This age group is not often given the needed attention when compared to the preschool age group in terms of nutritional assessment [25]. This is evident in the absence of this age group in Nigeria national nutritional survey.

There is evidence that catch-up growth can occur beyond preschool age [14]. Malnourished school children tend to have delayed bone age (maturational delay), which with adequate nutritional interventions, they may catch up during the pubertal period (second window of opportunity) [23, 26, 27]. This first window of opportunity occurs during the preschool age. Previous studies on malnutrition in Ebonyi State and in Abakaliki metropolis were among preschool children [2, 13].

Therefore, knowing the prevalence of malnutrition, particularly undernutrition and tackling the underlying factors will improve the overall welfare (nutrition wise) of school-age children in Abakaliki metropolis and increase their adult productivity and contributions to the Gross National Product. We conducted this study to determine the nutritional status of school age children in the Abakaliki metropolis.

## Methods

### Study area

The study was conducted in Abakaliki metropolis, Ebonyi State. The metropolis is made up of two Local Government Areas (LGAs) namely Abakaliki and Ebonyi LGAs. The metropolis is not entirely urban, some parts are still rural and are mainly occupied by indigenes. The combined population of both LGAs as at 2006 census was 276,909 [28]. There were 171 public primary schools and 85 private primary schools in the two LGAs. The population of primary school pupils in the metropolis was 67,236 (32,922 males and 34,314 females) according to the State Ministry of Education in 2015. The population of pupils in public schools was 54,902 while that of private schools was 12,334.

### Study design and population

We conducted a descriptive cross-sectional study among apparently healthy school children aged 6 to 12 years. Children who had history of chronic illnesses (like heart disease, asthma, sickle cell anaemia, chronic kidney disease, tuberculosis, skeletal abnormalities like scoliosis, kyphosis, genu varum and paralytic polio, Crohn’s and Celiac disease) and those who have been on steroid for up to 3 months were excluded.

### Sample size and sampling technique

We estimated the minimum number of children to be studied with the formula [29] below. The prevalence of stunting among preschool children was 20.6% (*P* = 0.206, Q = 1–0.206) in a recent national survey in Ebonyi State [13], Z the standard normal deviate corresponding to a 2 sided level of significance of 5% was 1.96 and precision of 3% (d = 0.03)

$$ n=\frac{Z^2 PQ}{d^2} $$

After adjusting for 10% non-response rate, a sample size of 780 pupils was calculated.

The pupils were selected using multistage sampling technique.

#### Stage 1

The list of all primary schools in Abakaliki metropolis was obtained from the Primary Education Board of State Ministry of Education. There were 171 public primary schools and 85 private primary schools in the metropolis. It was stratified into public and private schools in their respective Local Government Areas. Five schools (four public and one private) were selected from each of the two Local Government Areas in the metropolis, using simple random sampling through balloting method. Thus, a total of ten schools were selected. (five from each LGA). The ratio of 4:1 used in the selection was based on the population of pupils in public to private primary schools in the metropolis (54,902 in public and 12,334 in private schools). A sample of 78 pupils was allocated equally to each of the ten schools selected.

#### Stage 2

The pupils in each of the ten schools selected above were stratified by their classes. In schools with more than one arm per class, one arm was randomly selected through balloting to participate in the study. The 78 pupils allocated to the school were distributed to the six-participating classes proportionately based on the class population.

#### Stage 3

In each selected arm above, the pupils were stratified by sex into males and females and proportionately included in the study using a ratio of 1:1. In each stratum (boys or girls), the pupils were arranged serially, a total number of pupil in each stratum was determined. The pupils were selected in each strata using systematic sampling technique. The sampling interval k was calculated (k = number of pupil in the stratum divided by the half the sample size allocated to the arm). The starting point was randomly selected by balloting. Every k^th^ pupil was selected for the study. This was continued till the number allocated per stratum and per arm(class) was attained.

### Data collection

We used a pretested, structured and interviewer-administered questionnaire to collect information from the participants and a checklist to record the anthropometric measurement. The anthropometry was taken during their breaks and after dismissal. Weight in kilograms was taken using digital SECA® weighing scale. The weight was recorded to the nearest 100 g (0.1 kg). Each child was measured twice and an average taken to minimise measurement errors. Measurements were taken with the pupils on sports wears to minimize error due to different texture and weight of clothing. Before commencing each day, the weighing scale was inspected and checked for accuracy and consistency by using a known 2 kg weight.

Height in meters was measured using SECA stadiometer, model 213 Hamburg August 2014. The height was recorded to the nearest 1 mm (0.1 cm). To ensure uniformity, the heads of the pupils were placed in Frankfort position with the shoulders, buttocks and heels touching the vertical stand when measuring height in each child. Weight and height of each child were entered accordingly into their checklists, which was checked for completeness at the end of each day. The body mass index was calculated using the formula;

$$ BMI=\frac{Weight}{(Height)^2} $$

Weight was measured in kilograms (Kg) and height in meters (M). The data collection period lasted for six weeks (September to November 2017).

### Ethical approval

Ethical clearance was obtained from the Research and Ethical Committee of Federal Teaching Hospital Abakaliki with approval number 10/11/2016–17/11/2016. Permission was sought from Ebonyi State Ministry of Education, Universal Basic Education Board for public schools and the Proprietors /Administrators of the private schools. Consent was obtained from the parents/guardians and assent obtained from the pupils before the study.

### Data management and statistical analysis

We used the WHO 2007 criteria for measuring malnutrition in school-age children and adolescent [8]. Underweight was assessed using weight for age, thinness using BMI for age and stunting using height for age measure. Z score of less than -2Z was classified as underweight, thin or stunted. Z score of less than -2Z to -3Z were classified as moderate while Z scores of less than -3Z were classified as severe. The Z score of ‘weight for age’, according to WHO 2007 growth chart ranged from 5 to 10 years because weight for age is inadequate for growth monitoring beyond 10 years as it does not distinguish between relative height and body mass [30]. Therefore, underweight was assessed using weight for age Z score for children less than 10 years. Beyond 10 years of age, BMI for age complemented height for age. Undernutrition was derived from either underweight, thinness, stunted or combination of these. Overnutrition was defined as BMI for age greater than + 1 Z score, overweight was defined as BMI for age greater +1Z and less than +2Z score and obesity was defined as BMI for age greater than +2Z score. The socio-economic classification of the parents was estimated using Oyedeji classification [31].

The information on the socio-demographic characteristics of the children, parents’ characteristics and anthropometric measures were examined for completeness and errors, entered and analyzed using the Epi Info version 7.2, CDC Georgia United State of America 2017. Descriptive statistics of the socio-demographic characteristics, dependent variables and other related variables were carried out using frequencies and proportion. We also estimate the 95% confidence interval of the proportions of the main outcome variables however were not able to estimate sampling weights. The results were presented in tables and charts.

## Results

A total of 780 pupils were recruited from ten (two private and eight public) schools. Seven hundred and fifty-one (751) pupils participated fully giving a response rate of 96.3%. Age group 10–12 years was most represented (38.6%). Majority (69.4%) of the fathers had educational attainment higher than primary education while 70% of mothers had more than primary education (Table 1).

**Table 1:** Socio-demographic characteristics of the pupils and their parents

Variable	Frequency (*n* = 751)	Percentage (%)
Gender		
Male	354	47.1
Female	397	52.9
Age group (in years)		
6 - < 8	200	26.6
8 - < 10	261	34.8
10–12	290	38.6
Type of school		
Public	595	79.2
Private	156	20.8
Place of residence		
Rural	203	27.0
Urban	548	73.0
Father’s education		
No formal education	35	4.7
Primary education	195	26.0
Secondary education	278	37.0
Tertiary education	243	32.4
Father’s occupation		
Professionals	47	6.3
Public servants	179	23.8
Teachers	25	3.3
Skilled workers	183	24.4
Traders	124	16.5
Farmers	154	20.5
Unskilled workers	35	4.7
Unemployed	4	0.5
Mother’s education		
No formal education	30	4.0
Primary education	195	26.0
Secondary education	329	43.8
Tertiary education	197	26.2
Mother’s occupation		
Professionals	28	3.7
Public servants	94	12.5
Teachers	62	8.3
Skilled workers	53	7.1
Traders	332	44.2
Farmers	115	15.3
Unskilled workers	29	3.9
Unemployed	38	5.0
Parents’ social class		
Class 1	81	10.8
Class 2	173	23.0
Class 3	266	35.4
Class 4	175	23.3
Class 5	56	7.5

**Fig. 1:**
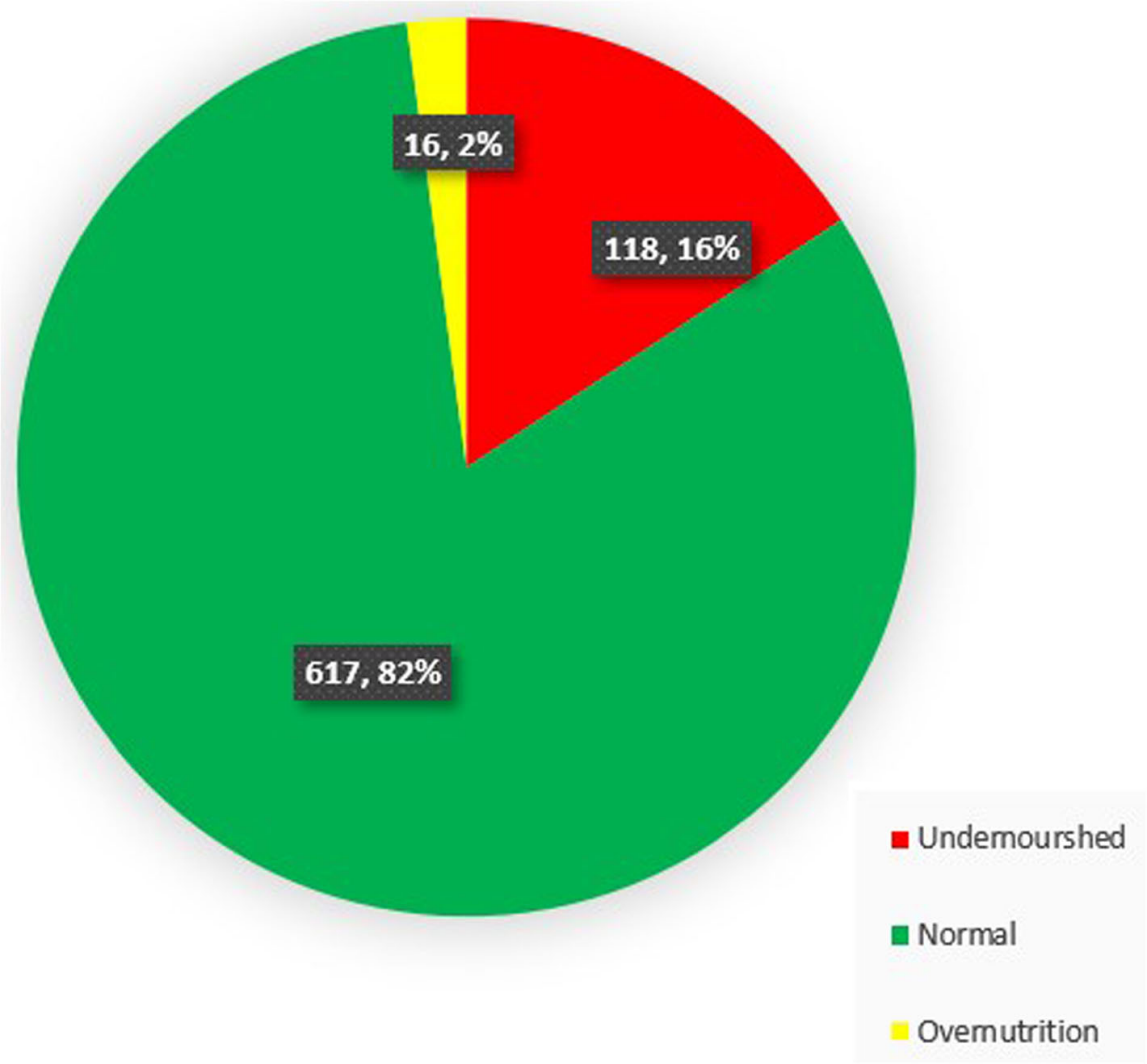
Prevalence of undernutrition in the study population

A total of 118 pupils (15.7%) were undernourished (either underweight, thin or stunted or had a combination of any of these). Two per cent of the study population (16 pupils) were over nourished (Fig. 1).

The prevalence of underweight among pupils aged 6 to 10 years was 8%. Thirty-four pupils (7%; 95%CI; 5.1–9.7) were moderately underweight while 5 pupils (1%; 95%CI: 0.4–2.4) were severely underweight (Table 2).

**Table 2:** Prevalence of underweight using weight for age Z score among the pupils aged 6–10 years disaggregated by gender, school type and place of residence

Variable	Normal (%)	Moderate underweight (%; 95%CI)	Severe underweight (%; 95%CI)
Overall status	445 (92.0)	34 (7.0; 5.1–9.7)	5 (1.0; 0.4–2.4)
Gender			
Male	213 (92.2)	16 (6.9; 4.3–11.0)	2 (0.9; 0.2–3.1)
Female	232 (91.7)	18 (7.1; 4.5–11.0)	3 (1.2; 0.4–3.4)
School ownership type			
Public	321 (89.2)	34 (9.4; 6.8–12.9)	5 (1.4; 0.4–3.2)
Private	124 (100.0)	0 (0; 0.0–3.0)	0 (0; 0.0–3.0)
Place of residence			
Urban	342 (93.4)	22 (6.0; 4.0–8.9)	2 (0.6; 0.2–2.0)
Rural	103 (87.3)	12 (10.2; 5.9–16.9)	3 (2.5; 0.9–7.2)

The prevalence of underweight was 7.8% among males [6.9 and 0.9% for moderate and severe underweight respectively] while among females the prevalence of underweight was 8.3% [7.1 and 1.2% for moderate and severe underweight respectively]. No pupil was found to be underweight among those attending private schools but 39 (10.8%) pupils [9.4% moderately underweight and 1.4% severely underweight] were underweight among pupils attending public schools. The prevalence of underweight among 115 pupils living in rural area was 12.7% (Table 2). In addition, the figure showed an overweight in eleven (1.4%) pupils and obesity in five (0.7%) pupils (Table 3).

**Table 3:** Prevalence of thinness using BMI for age Z score among the pupils disaggregated by gender, school ownership type and place of residence

Variable	Severe thinness (%; 95%CI)	Moderate thinness (%; 95%CI)	Normal (%)	Overweight (%, 95CI)	Obese (%; 95%CI)
Overall status	6 (0.8;0.4–1.7)	48 (6.4;4.9–8.4)	681 (90.7)	11 (1.4; 0.8–2.6)	5 (0.7; 0.3–1.5)
Gender					
Male	4 (1.1; 0.4–2.9)	27 (7.6; 5.3–10.9)	318 (89.8)	3 (0.9;0.3–2.5)	2 (0.6;0.2–2.0)
Female	2 (0.5;0.1–1.8)	21 (5.3; 3.5–8.0)	363 (91.4)	8 (2.0; 1.0–3.9)	3 (0.8; 0.3–2.2)
School type					
Public	6 (1.0; 0.5–2.2)	45 (7.6; 5.7–10.0)	539 (90.6)	5 (0.8; 0.4–2.0)	0 (0; 0.0–0.6)
Private	0 (0; 0.0–2.4)	3 (1.9; 0.7–5.5)	142 (91.0)	6 (3.9; 1.8–8.1)	5 (3.2; 1.4–7.2)
Place of residence					
Urban	2 (0.4; 0.1–1.3)	23 (4.2; 2.8–6.2)	507 (92.5)	11 (2.0; 1.1–3.6)	5(0.9; 0.4–2.1)
Rural	4 (2.0; 0.8–5.0)	25 (12.3; 8.5–17.6)	174 (85.7)	0 (0; 0.0–1.9)	0 (0; 0.0–1.9)

Fifty-four (7.2%) of the study population were thin whereas, 1.4% were overweight and 0.7% were obese. The proportion of thinness was slightly higher among males (8.7%) compared to females (5.8%) The proportion of thinness was higher among pupil attending public schools (8.6%) compared to those attending private schools (1.9%). The prevalence of thinness was much higher among the pupils residing in rural areas (14.3%) compared to those residing in urban areas (4.6%) (Table 3).

The prevalence of stunting among the pupils was 9.9 (Table 4). The prevalence of severe stunting was more among the male participants. The prevalence of stunting was found to be higher among pupils attending public schools (11.8%) compared to those attending private schools (2.5%). The prevalence of stunting was 19.3% (15.8 and 3.5% for moderate and severe stunting respectively) among the pupils residing in rural areas and 5% (4.7 and 0.3% for moderate and severe respectively) among the pupils living in urban areas of the metropolis (Table 4).

**Table 4:** Prevalence of Stunting using HAZ Z score among the pupils disaggregated by gender, school ownership type and place of residence

Variable	Normal (%)	Moderate stunting (%; 95%CI)	Severe stunting (%, 95CI)
Overall status	677 (90.1)	59 (7.9;6.1–10.0)	15 (2.0; 1.2–3.3)
Gender			
Male	319 (90.1)	25 (7.1; 4.8–10.2)	10 (2.8; 1.5–5.1)
Female	358 (90.1)	34 (8.6; 6.1–11.7)	5 (1.3; 0.5–2.9)
School type			
Public	525 (88.2)	56 (9.4; 7.3–12.0)	14 (2.4; 1.4–3.9)
Private	152 (97.4)	3 (1.9; 0.7–5.5)	1 (0.6; 0.1–3.5)
Place of residence			
Urban	515 (94.0)	26 (4.7; 3.3–6.9)	7 (0.3; 0.6–2.6)
Rural	164 (80.8)	32 (15.8; 11.4–21.4)	7 (3.5; 1.7–6.9)

## Discussion

The prevalence of undernutrition among school age children in the metropolis was high. The high value may be as a result of significant rural population that made up the metropolis where most cases of undernutrition in this study were found. The finding suggests that much higher figures might be obtained beyond the borders of the metropolis in the State. Thus, if measures are not put in place to reduce the level of undernutrition, the prevalence of undernutrition might worsen over time, in view of Nigeria’s dwindling economy which has put many families into extreme poverty and hunger. This result suggests that undernutrition is also prevalent among school-age children. This age group need to be considered in programing interventions for undernutrition in order to get a holistic solution to the problem of undernutrition. The prevalence of undernutrition was, however, lower than those reported in some earlier studies [6, 32].

The prevalence of underweight was 8% with 1% of the pupils being severely underweight. This finding was high considering that the study area is largely an urban population and that the age group studied were not completely dependent on adults for their nutrition compared to the under-fives. Underweight is a measure of previous and recent weight loss or failure to gain weight [4] which is largely due to shortage in food supply, availability or intake. A relationship has been established between underweight and food insecurity [33]. Therefore, this finding showed a possible underlying challenge of food security in the study population.

There was no national or state prevalence of underweight among school-age population for comparison. However, the 2015 National Nutrition Health Survey reported a higher underweight prevalence of 11.5% among the preschool population in Ebonyi State [13]. The high prevalence of underweight among the school-age children in this study further underscores the need for interventions among this age group in the metropolis.

The findings of studies done in other parts of the country among the school-age children varied greatly. The prevalence of underweight in our study fell within the range reported in other studies done in Southeast Nigeria [14, 34–36]. Lower prevalence has however been reported in a neigbouring state among urban dwellers [34, 35]. Other studies in Nigeria [5, 32, 37, 38], Ghana [22], Ethiopia [6, 7] and India [16] have reported higher prevalence of underweight.

The prevalence of underweight was higher among pupils residing in the rural areas of the metropolis compared to those residing in urban areas though not statistically significant. Similar observation had been reported in previous studies [38–40]. In this study, underweight was not found in pupils attending private schools, however, in Uyo, 27.3% of the pupils attending private schools were underweight [41]. The higher maternal level of education of pupils in private schools found in this study may be a reason for this observation. Maternal educational status contributes reasonably to the family’s socio-economic status which invariably affects the nutrition and health of their children [42]. The limited space for play and long sitting periods for learning (sedentary lifestyle) and provision of lunch or break time snacks to pupils in private schools by their parents may also explain the absence of underweight among the pupils in private schools as these practices are uncommon in public schools.

Thinness is a measure of acute undernutrition. The prevalence of thinness in this study indicates that school-age children in Abakaliki are facing acute shortage in food supply or intake. The prevalence of thinness varied greatly from place to place. The study prevalence was higher than that reported in Enugu [34] and Jos [37] but lower than that reported in Sagamu [5] all in Nigeria. The lower prevalence of thinness reported in Enugu may be because their sample was drawn mainly from private schools as opposed to this study where most of its participants were from public schools.

Prevalence of thinness in this study was observed to be more among males though not statistically significant. Similar male preponderance had been observed [3, 32]. The prevalence of thinness was higher among pupils in public schools compared to those in private schools. This may not be unconnected to the differences in the socioeconomic characteristics of their parents. Parents of higher socioeconomic class are more likely to send their children to private schools compared to those of lower socioeconomic status. This assertion was further strengthened by higher prevalence of overweight and obesity among the pupils attending private schools.

We observed a low prevalence of overnutrition. Our result was much lower than those reported in elsewhere in Nigeria, Ghana and Vietnam [34, 43–45]. Overweight was observed more in girls and among pupils in private schools. This preponderance of overweight among pupils in private schools compared to those in the public schools has been reported [43, 46]. The observation may be due to the effect of estrogen on fat accumulation in females. Parents in the high socioeconomic class are more likely to send their children to private school and the children in private schools are more exposed to sedentary lifestyle compare to public schools. The relationship between high socioeconomic class of the parent of children attending private schools, sedentary lifestyle and overweight has earlier been reported [45, 47]. Over nutrition was not found among pupils residing in the rural areas. The implication of this finding is that any intervention to address thinness in schools should be segmented and appropriately targeted especially in an emerging city. Such interventions need to be comprehensive in tackling thinness on one hand and overweight/obesity on the other hand.

Stunting was the most prevalent form of undernutrition found in this study. This level of stunting in a metropolis is of public health importance, considering its implications on child growth and development especially for the girl child. The effects of stunting on the girl child transcends one generation due to its consequences on pregnancy and childbirth. Considering this transgenerational effect of stunting (a stunted mother is more likely to beget a low birth weight baby who becomes stunted and the cycle continues), there is urgent need to reverse this trend.

The main food sources in Ebonyi State being yam, rice and cassava may explain out this level of chronic undernutrition. These food types are mainly energy giving foods and are usually prepared with little or no protein (body building foods): eggs, fish or meats. The relationship between lack of food diversity and stunting had earlier been reported [48]. This raise the need for dietary diversification in tackling the effect of undernutrition in children. To achieve this there is need for adequate sensitization of the mothers especially in rural areas where the literacy level is low.

The same prevalence of stunting as reported here was reported in an earlier study in Abakaliki metropolis among the preschool children [2]. This probably suggests that either there were no interventions to address stunting or the interventions have not yielded the expected results. A lower prevalence of were reported in two different studies in urban areas of Enugu [34, 35] while in Onitsha, Anambra State, a much higher prevalence was reported [36]. The high prevalence observed in Anambra could be related to the study area which was known to harbour people with low income and this could affect their food choice and health-seeking behaviour [49].

In North central Nigeria, the prevalence (10.3%) reported in rural community in Jos compared well with the current study prevalence [50]. A much higher prevalence has been reported elsewhere in Nigeria [5, 18, 32] and in Africa [6, 7, 40, 51].

## Conclusion

Undernutrition was prevalent among school age children in Abakaliki metropolis. The prevalence was more among pupils in public schools compare to private school, those living in rural areas compared to the urban. This suggests that nutritional interventions should be targeted based on the need of the various subpopulation for effective control of the malnutrition.

## Data Availability

The datasets used and/or analyzed during the current study are available from the corresponding author on reasonable request.

## Abbreviations

BMI: Body mass index
CDC: Centers for Disease Control and Prevention
GDP: Gross domestic product
GNR: Global nutrition report
LGA: Local Government Area
WHO: World Health Organization
